# Multiple Chromoanasynthesis in a Rare Case of Sporadic Renal Leiomyosarcoma: A Case Report

**DOI:** 10.3389/fonc.2020.01653

**Published:** 2020-08-19

**Authors:** Kirill Igorevich Anoshkin, Kristina Olegovna Karandasheva, Kristina Mikhaylovna Goryacheva, Denis Valer’yevich Pyankov, Philipp Aleksandrovich Koshkin, Tatiana Vladimirovna Pavlova, Alexandr Nikolaevich Bobin, Evgeniy Valer’yevich Shpot, Yaroslav Nikolayevich Chernov, Andrei Zinov’yevich Vinarov, Dmitry Vladimirovich Zaletaev, Sergei Ivanovich Kutsev, Vladimir Viktorovich Strelnikov

**Affiliations:** ^1^Research Centre for Medical Genetics, Moscow, Russia; ^2^I.M. Sechenov First Moscow State Medical University, Moscow, Russia; ^3^Genomed Ltd., Moscow, Russia; ^4^N.N. Burdenko Main Military and Clinical Hospital, Moscow, Russia

**Keywords:** leiomyosarcoma, renal, chromoanagenesis, chromoanasynthesis, MDM2, microarray, NGS, chromosomal instability

## Abstract

We present the genetic profile of kidney giant leiomyosarcoma characterized by sequencing of 409 cancer related genes and chromosomal microarray analysis. Renal leiomyosarcomas are extremely rare neoplasms with aggressive behavior and poor survival prognosis. Most frequent somatic events in leiomyosarcomas are mutations in the TP53, RB1, ATRX, and PTEN genes, chromosomal instability (CIN) and chromoanagenesis. 67-year-old woman presented with a right kidney completely replaced by tumor. Immunohistochemical reaction on surgical material was positive to desmin and smooth muscle actin. Molecular genetic analysis revealed that tumor harbored monosomy of chromosomes 3 and 11, gain of Xp (ATRX) arm and three chromoanasynthesis regions (6q21-q27, 7p22.3-p12.1, and 12q13.11-q21.2), with MDM2 and CDK4 oncogenes copy number gains, whereas no copy number variations (CNVs) or tumor specific single nucleotide variants (SNVs) in TP53, RB1, and PTEN genes were present. We hypothesize that chromoanasynthesis in 12q13.11-q21.2 could be a trigger of observed CIN in this tumor.

## Introduction

Leiomyosarcomas (LMSs) of the kidney are very rare renal tumors that account for 0.12% of all renal malignancies (1). Most often leiomyosarcomas of the kidney are found in females with a mean age of 50–60 years and have poor survival prognosis (1, 2). Despite the high representation among renal sarcomas (50–60%), the information about LMS in the kidney is limited. Most commonly renal LMSs originate from smooth muscles of kidney veins, but can also arise from renal capsule, renal pelvis, blood vessels, and calyxes (3).

Current studies, focused on the genetic profiling of LMS aggregate tumors from different localizations (lower extremity, trunk, uterus, or retroperitoneum) where in the latter case, specific locations of the tumor are not specified (4–7). Leiomyosarcomas of any location predominantly have mutations in *TP53*, *RB1*, *ATRX*, and *PTEN* or copy number changes involving these genes and also harbor multiple chromosomal rearrangements including chromoanagenesis (8).

As far as we know, the single research was published, in which authors aimed to study the cytogenetic profile of renal LMS, and showed hypotetraploid karyotype in tumor material (9). Yet, there are no published studies with more extensive genetic approach on leiomyosarcoma of the kidney. Here we present a rare case of leiomyosarcoma of the right kidney with radiographic findings, microscopic and immunohistochemical examination, molecular genetic profiling of 409 tumor related genes and chromosomal microarray analysis.

## Case Presentation

A 67-year-old woman with enlarged abdomen, dull pain in the lumbar region on the right, palpitation and moderate weakness was admitted to the Urological clinic department of I.M. Sechenov First Moscow State Medical University in July 2014. The patient reported no relevant clinical family history. The patient signed informed agreement to undergo diagnostic procedures and treatment, as well as to participate in the study, and for the presentation of clinical and molecular data in scientific and medical literature. This case report was approved by the local Ethics Committee at the Research Centre for Medical Genetics, Moscow, Russia.

**FIGURE 1:**
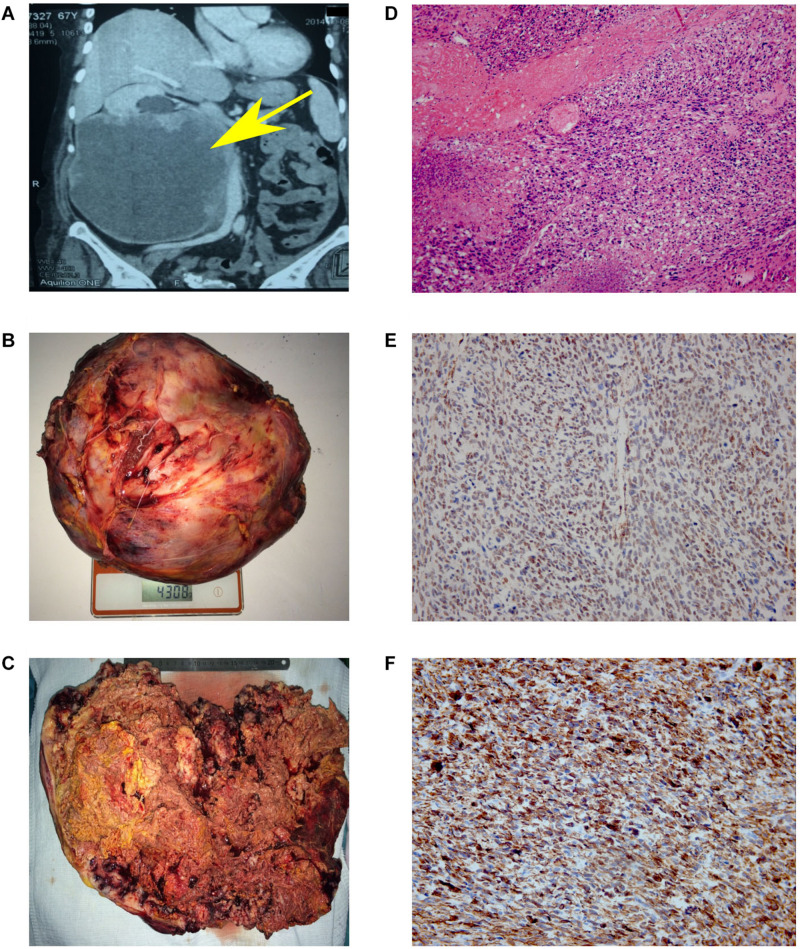
Results of MSCT, macro and microscopy. **(A)** MSCT of the abdominal cavity with contrast. Neoplasm in the middle and lower segments’ projection of the right kidney, in size of 147 × 190 × 188 mm, which unevenly accumulated a contrast agent (from 35 units N. to 50–90 units N.). No signs of invasion of the vascular pedicle were noted. **(B)** Back table examination. Right kidney with a tumor measuring 300 × 253 × 150 mm (4308 g). **(C)** In the section, the right kidney is completely replaced by yellow-brown tumor masses. **(D)** Hematoxylin and eosin (H&E)-stained slide. The tumor is represented by interwoven bundles of stretched cells with enlarged hyperchromic nuclei, foci of necrosis and hemorrhages. Mitoses are also observed (original magnification ×100). **(E)** Immunohistochemical reaction with antibodies to SMA (original magnification ×200). **(F)** Immunohistochemical reaction with antibodies to desmin (original magnification ×200).

The patient underwent an ultrasonic examination that revealed a mass in the right kidney with a size of 176 × 164 mm. Multi-slice spiral computed tomography showed that right kidney was displaced upward, rotated anteriorly because of spherical soft tissue mass in the middle and lower segments of the kidney with a size 206 × 186 × 188 mm and clear contours in the structure of which arterial vessels were determined (Figure 1A). Lesion extended into the sinus of the right kidney, ingrew in ureter at the ureteropelvic junction and more distal and encircled pelvis. Lesion also lied tightly next to psoas major muscle and inferior vena cava. The pyelocaliceal system of the right kidney was expanded: a calyx and a pelvis were 30 mm and 45 × 28 mm in size, respectively. The mass unevenly accumulated a contrast agent. Excretion of the contrast agent by the right kidney was absent.

### Treatment

Based on the obtained diagnostic data, the multidisciplinary board was held, implying the participation of surgical oncologist, oncologist, pathomorphologist, a specialist in radiation diagnostics and urologist. The nephrectomy of the right kidney and regional lymphadenectomy was performed. During the operation, it was noted that the inferior vena cava from the adrenal gland to aortic bifurcation is involved in the tumor process. The invasion into the liver was not observed. The resected tumor had a size of 300 × 253 × 150 mm and 4 kg of weight (Figure 1B). Incision shows that the right kidney was completely replaced by tumor mass (Figure 1C).

### Outcome

Despite the successful surgery, the follow up was not possible by the reason of patient’s death in post-operative period. The cause of death was not related to nephrectomy.

### Microscopy and Immunohistochemistry

The microscopic examination of the tumor revealed plexiform bundles of spindle-shaped cells with large hyperchromatic nuclei, among which multinucleated cells were found. High mitotic activity (17 mitoses per 10 fields with magnification ×400) and foci of necrosis were observed (Figure 1D). The immunohistochemistry showed positive reaction for smooth muscle actin (Figure 1E) and desmin (Figure 1F) and negative reaction for CD99, CD43, CD117, S-100, MCK/PCK, and DOG-1. Taking into account the histological and immunohistochemical data, the changes correspond to Grade 3 kidney leiomyosarcoma (overall score of 6 points: tumor differentiation, 3; mitotic activity, 2; necrosis, 1) according to FNCLCC gradation of soft tissue tumors (10).

## Materials and Methods

### DNA Extraction

DNA was extracted from the formalin-fixed, paraffin-embedded tumor tissue by using GeneRead DNA FFPE kit (Qiagen, Germany), and from whole peripheral blood using standard phenol-chloroform extraction protocol.

### DNA Sequencing

DNA sequencing was performed using Ion AmpliSeq targeted amplification technology and AmpliSeq Comprehensive Cancer Panel (Thermo Fisher Scientific, United States). The cancer-specific primer panel provides exon coverage of 409 oncogenes and tumor suppressor genes.

### Bioinformatic Analysis

The bioinformatic workflow for sequencing data analysis was based on Torrent Suite software (version 5.10.1). CNVs were estimated using the ONCOCNV (11) package (version 6.9).

### Chromosomal Microarray Analysis

For the detection of chromosomal aberrations, we used an SNP-array CytoScan HD (Thermo Fisher Scientific, United States) on GeneChip Scanner 3000 7G System (Applied Biosystems, United States) following the manufacturer’s recommendations. Data were analyzed with Chromosome Analysis Suite software (Affymetrix). All copy number alterations were manually reviewed. To detect and discern chromoanasynthesis we used criteria that were previously described (12–14): more than five breakpoints with clustered distribution of the segments with normal copy number interspersed with copy number gains.

## Results

### Next Generation Sequencing

Next generation sequencing was performed on gDNA of whole blood and FFPE tumor tissue sample. Median of read coverage in tumor and blood samples was 393× and 371×, respectively. No mutations associated with any genetic syndrome were found. We did not find any pathogenic SNVs in the most commonly mutated in LMS genes *TP53*, *RB1*, *ATRX*, and *PTEN*, as well as in less commonly mutated, *ATM* and *EGFR2* (15). Several SNVs previously reported as common for LMS (7) in the *KLF6* (NC_000010.10:g3824388_3824389insG), *WAS* (NC_000023.10:g.48547111_48547112insC), *AKT1* (NC_000014.8:g.105238770_105238771insC), and *GPR124* (NC_000008.10:g.37692704G>A) genes were also present in our sample, but we have observed low frequency of alternative alleles and sequencing strand bias for these variants. We have also revealed these SNVs in other samples that were analyzed in our laboratory, also with low frequency of alternative allele and with strand bias. Since we used the same targeted amplification and sequencing technology and the same cancer-specific primer panel (AmpliSeq Comprehensive Cancer Panel, Thermo Fisher Scientific, United States) as in work of Rao et al. (7), we suggest that these SNVs are merely sequencing artifacts.

### Chromosomal Microarray Analysis

**FIGURE 2:**
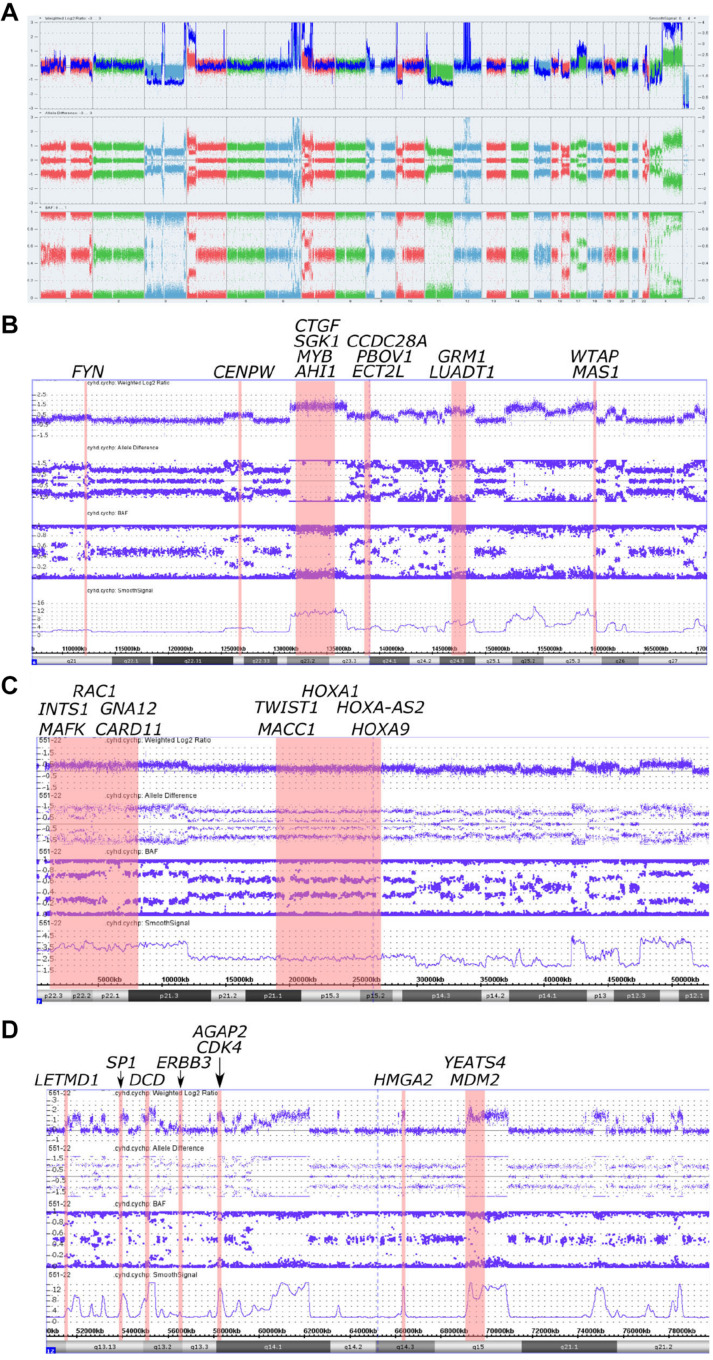
Copy number alterations in whole genome view summary and regions with chromoanasynthesis presented by weighted Log2 Ratio, allele difference, B-Allele Frequency (BAF) and smooth signal. **(A)** Whole Genome View summary. **(B)** Chromoanasynthesis in 6q21-q27 region that harbors 13 oncogenes including *MYB*. **(C)** Chromoanasynthesis in 7p22.3-p12.1 region that harbors 10 oncogenes including *MACC1*. **(D)** Chromoanasynthesis in 12q13.11-q21.2 region that harbors nine oncogenes including *MDM2* and *CDK4* in q13.3 and q15 bands, respectively.

By using chromosomal microarray analysis (CMA), we have revealed numerous chromosomal aberrations in the tumor sample. The tumor carried a vast number of gains and losses of fractions of chromosomes, and also monosomy of chromosomes 3 and 11 (Figure 2A and Supplementary Figures 1, 2A,B) which in complex refers to chromosomal instability (CIN). Gains were seen in 1p22.2-p21.3, 2p25.3-p24.3, 3q28-q29, 4p16.3-p13, 4q34.2-q34.3, 9p24.3-p23, 10p15.3-p15.2, 11p15.4, 17q12-q25.3, 19p13.3, 22q12.1-q13.1, and Xq. Heterozygous losses were observed in 1q43-q44, 7q35, 10p15.3, 10p15.1-p12.1, 15q, 16q, 17q25.3, 18q23, 19q, 22q12.1, 22q13.2-q13.33, and Xp (Figure 2A and Supplementary Figure 1). These results were generally confirmed by ONCOCNV analysis of the NGS data (Supplementary Figure 3). Fifteen short regions of homozygous losses varying from 8 nucleotides to 136 kilobases were identified (Supplementary Table 1). Two regions with homozygous losses affected genes *PTCHD3* [arr(GRCh37)p12.1(27625952_27688513)x0]) and *IL3RA* [arr(GRCh37)Xp22.33(1459624_1460944)x0] in 3′UTR and 5′UTR, respectively. We did not observe copy number variations in regions that harbor *PTEN*, *TP53*, or *RB1* genes, but gain of Xq arm where *ATRX* (Xq21.1) gene is located was observed (Supplementary Figure 4).

In addition, we discovered three regions (6q21-q27, 7p22.3-p12.1, and 12q13.11-q21.2) with at least 13 switches alternating different copy gains with normal copy state that refers to chromoanasynthesis (Figures 2B–D). These regions harbor 31 oncogenes (Table 1) according to ONGene database (16) among which are *MYB* and *FYN* in 6q region (Figure 2B), *MACC* in 7p region (Figure 2C), *MDM2* and cyclin dependent kinase 4 (*CDK4*) (Figure 2B and Supplementary Figures 5A,B) in 12q region. Analysis of the location of breakpoints in chromoanasynthesis has revealed that in total 60 genes, including 2 oncogenes (*GLI3*, *HMGA2*), 6 tumor suppressor genes (17) and *SP1* transcription factor gene were found to be truncated by chromoanasynthesis (Table 1).

**TABLE 1:** Genes that are located in regions with chromoanasynthesis divided into groups of oncogenes and tumor suppressor genes according to Ongene^16^ and TSGene 2.0^17^ databases, as well as truncated genes which were observed in breakpoints of chromoanasynthesis.

	Chromoanasynthesis in region		
	6q21-q27	7p22.3-p12.1	12q13.11-q21.2
Amplified oncogenes	*MAS1, AHI1, GRM1, SGK1, MYB, CCDC28A, CENPW, FYN, ECT2L, WTAP, PBOV1, LUADT1, CTGF*	*CARD11, GNA12, HOXA1, HOXA9, HOXA-AS2, INTS1, MACC1, RAC1, TWIST1, MAFK*	*MDM2, CDK4, DCD, HMGA2, AGAP2, LETMD1, YEATS4, ERBB3*

	**Truncated genes in observed regions with chromoanasynthesis**		

Oncogenes		*GLI3*	*HMGA2*
Tumor suppressor genes	*FOXO3, IGF2R, PACRG, PLAGL1*	*SFRP4, POU6F2*	
Other genes	*EPM2A, RMND1, UNC93A, TCP10L2, CCDC170, DACT2, EPB41L2, FAM120B, PHACTR2, PSMB1*	*NPSR1, EPDR1, TNS3, HOXA11, NPSR1-AS1, GPR141, ELMO1, TRA2A, BBS9, DPY19L1P1, AVL9, FAM221A, LINC01162, HOXA13, CAMK2B, SEPT7P2, STK31, YAE1D1*	*KRT4, RPSAP52, PCED1B, ZDHHC17, WIBG, SP1, SMUG1, SCN8A, RAP1B, R3HDM2, PTPRQ, PTPRB, OSBPL8, OS9, OR6C7P, NAV3, MDM1, KRT86, KRT83, KRT7, KRT121P, GLIPR1L1, FAM19A2, CAPS2*

## Discussion

Here we report a rare case of leiomyosarcoma of the kidney with multiple chromoanasynthesis and monosomy of 3 and 11 chromosomes. According to recent studies, LMSs of any location are known to be tumors hallmarks of which are various types of mutations in *TP53* (17p13), *RB1* (13q14), *PTEN* (10q23), or *ATRX* (Xq21.1) genes (4–6, 15, 18, 19). SNVs are the most common types of mutation occurring in 71% in LMSs (4), but in the case presented here, DNA sequencing of 409 tumor related genes did not reveal any pathogenic SNVs in *TP53*, *RB1*, *PTEN*, and *ATRX* or any other of the analyzed genes. Few shared SNVs observed by Rao et al. (7) were also revealed in our case, however, our in-house database classifies them as sequencing artifacts intrinsic for the sequencing method used.

By using CMA we found that tumor harbored a vast amount of copy number aberrations (>20) including monosomy of 3 and 11 chromosomes, which are signs of CIN (20–22) (Figure 2A and Supplementary Figures 1, 2A,B, 3). Previously cytogenetic research of leiomyosarcomas revealed that there is a correlation between the quantity of changes and size of tumors (23). It was noticed that tumors larger than 5 cm had a mean of copy number changes of 13 (23). Additionally, gains of 1q, 5p, 6q, and 8q were also correlated with larger tumors (23). Abundance of CNVs observed in our sample, as well as 1q and 6q gains, support this observation. With regards to the correlation of tumor size and copy gains, chromoanasynthesis discovered in 6q arm that harbors 13 oncogenes including *MYB*, could also theoretically correlate with large size of the tumor (Figure 2B). *MYB* had previously been suggested a likely candidate oncogene involved in LMS pathogenesis (23). We have also observed monosomy of 3 and 11 chromosomes, which is not a common event in leiomyosarcoma (24–26) (Figure 2A and Supplementary Figures 1, 2A,B, 3). Studies report that karyotypes in LMS are not stable and harbor various and numerous CNVs (4, 5, 21, 23). Aberrations in leiomyosarcomas mostly occur in regions 17p13, 13q14, 10q23, affecting *TP53*, *RB1*, and *PTEN* genes; however, other less recurrent aberration were also found (4, 5, 23). Aberration in our sample did not occur in bands 17p13, 13q14, 10q23, whereas gain was detected in the long arm of the X chromosome, where the *ATRX* (q21.1) gene is located (Figure 2A and Supplementary Figure 4).

Additionally, with CMA we have determined three regions with chromoanasynthesis, which is one type of chromoanagenesis (Figures 2B–D). Chromoanagenesis is a massive complex of genomic and chromosomal aberrations characterized by tens to hundreds of simultaneous locally clustered DNA rearrangements, in which from one to multiple chromosomes can be involved (14, 27). Chromoanagenesis is considered to be a rare (5%) but crucial event in the pathogenesis of cancer (8, 28). However, in a recent research conducted by Cortés-Ciriano et al. (8) chromoanagenesis (chromothripsis) was identified in 29% of analyzed cancer samples, although the frequency varied markedly across cancer types, with more than 50% in soft tissue sarcomas and leiomyosarcomas (8).

Chromoanagenesis groups three major distinct phenomena: chromothripsis, chromoanasynthesis, and chromoplexy and other rare chromothripsis-like events (14, 27, 29, 30). The first discovered chromoanagenesis event was chromothripsis (*chromas* – chromosome and *thripsis –* shattering into pieces) described by Stephens et al. (31), which is often used nowadays to describe all types of such phenomenon (8, 32). With respect to the term and to authors using it, we will use chromoanagenesis instead of chromothripsis to further combine these phenomena. Rearrangements that are caused by chromoanagenesis can impact genome in various ways, such as pathogenic gene fusion, truncation of haploinsufficient or tumor suppressor genes in breakpoints, or the formation of double minute chromosomes containing oncogenes that might gain increased expression through an increase in the number of their copies (12, 14, 33–37).

The key difference between chromoanasynthesis and chromothripsis is that chromoanasynthesis harbors copy gains in addition to losses, whereas chromothripsis has almost complete absence of gains (14, 38). Typically, chromoanasynthesis occurs in one or few chromosomes (12–14). It is proposed that the cause of chromoanasynthesis is a replication-based mechanism, either microhomology-mediated break-induced replication (MMBIR), or serial fork stalling and template switching (FoSTeS) (14). In these models, lagging DNA strand serially disengage and goes to another nearby template; a series of microhomology-dependent template and switching events occur resulting in chromoanasynthesis (39).

Several studies show that such replication-based models may occur in micronuclei (40–42). Crasta et al. (40) performed an experiment which showed that p53 knockout results in an increasing number of micronuclei with evidence of double strand breaks in them. It was also demonstrated that fragmentation and subsequent assembly of chromatids localized in micronuclei can initiate MMBIR process (41). Research shows that disruption of p53 pathway, primarily in *TP53* gene, are associated with chromoanagenesis in pediatric cancers, and can be a trigger for CIN and numerical or structural aneuploidies (37, 43). However, Cortés-Ciriano et al. (8) showed that only 38% samples with inactivating *TP53* mutations demonstrate chromoanagenesis, whereas 60% of samples with chromoanagenesis show no mutation in *TP53* nor *MDM2* amplification. In our case chromoanasynthesis in 12q region affects *MDM2* oncogene and *CDK4*, with their fourfold copy number increase (Supplementary Figures 5A,B). These genes play key roles in cell cycle and are often co-amplified in sarcomas. They are also targets that are approved or clinically tested for therapy (44). Furgason et al. (45) and Garsed et al. (46) in their studies suggest that it is very likely that chromoanagenesis occurring in chromosome 12q region, that harbors *MDM2* and *CDK4*, cause the derivation of double minute chromosomes through disruption of p53 pathway.

We have also found that chromoanasynthesis in 6q21q27 region harbors *MYB* oncogene (Figure 2B). *MYB* oncogene is involved in the regulation of cell proliferation, differentiation and apoptosis (47). Highly expressed *MYB* was shown in leukemias, hereditary BRCA1 breast cancer and colon cancer (47–50).

In the 7p arm chromoanasynthesis region, we detected *MACC1* gene gain (Figure 2C). Metastasis associated in colon cancer 1 (MACC1) is an oncogene that regulates HGF/c-Met pathway that is known to play significant role in carcinogenesis by suppressing apoptosis and facilitating migration and invasion of cancer cells (51). *MACC1* overexpression results in promotion of cell proliferation (52).

We have described a spectrum of molecular genetic alterations in a rare case of giant renal leiomyosarcoma, among which chromoanasynthesis in 12q13.11-q21.2 which harbor crucial genes that are involved in cell cycle, *MDM2* and *CDK4*, may be speculated to trigger CIN events including chromoanasynthesis in 6q21-q27 and 7p22.3-p12.1. Involvement of p53 pathway deregulation in the development of such massive chromosomal aberrations has been described in multiple studies (8, 37, 40, 41, 43, 45, 46, 53). As for chromoanasynthesis in 12q13.11-q21.2 itself, it could be caused by a diversity of cellular events underlying replication defects that can initiate genome instability (54). While this explanation is hypothetical, accumulation of well characterized cases of rare tumors will potentiate identification of their molecular etiology and pathogenesis with the perspective of better diagnostics and treatment.

## Data Availability Statement

The datasets presented in this study can be found in online repositories. The names of the repository/repositories and accession number(s) can be found below: https://www.ncbi.nlm.nih.gov/geo/, GSE151766; https://www.ncbi.nlm.nih.gov/, PRJNA635906.

## Ethics Statement

The patient signed informed agreement to undergo diagnostic procedures and treatment, as well as to participate in the study, and for the presentation of clinical and molecular data in scientific and medical literature.

## Author Contributions

KA and VS analyzed the results. KK performed the bioinformatics analysis of sequence data. KG, ES, and YC performed the clinical investigations. DP and PK performed the chromosomal microarray analysis. TP and AB performed the microscopy and immunohistochemistry. AV, DZ, and SK administered the project. All authors reviewed the manuscript.

## Conflict of Interest

DP and PK were employed by the company Genomed Ltd. The remaining authors declare that the research was conducted in the absence of any commercial or financial relationships that could be construed as a potential conflict of interest.
