# Supplementary material for: Multiple Chromoanasynthesis in a Rare Case of Sporadic Renal Leiomyosarcoma: A Case Report
Source: Front Oncol. 2020 Aug 19;10:1653. doi: 10.3389/fonc.2020.01653 (PMC7466669; doi:10.3389/fonc.2020.01653)
Supplement: Supplementary file 1 [file Data_Sheet_1.PDF]

Supplementary Material

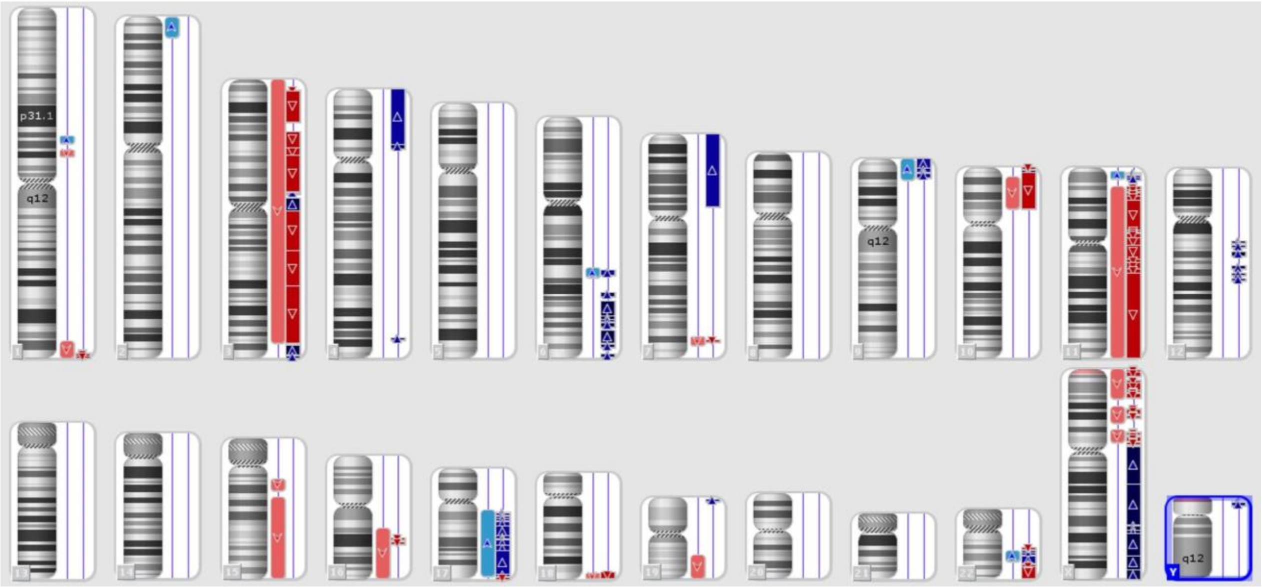

Supplementary Figure 1. Karyoview of analyzed renal leiomyosarcoma sample.

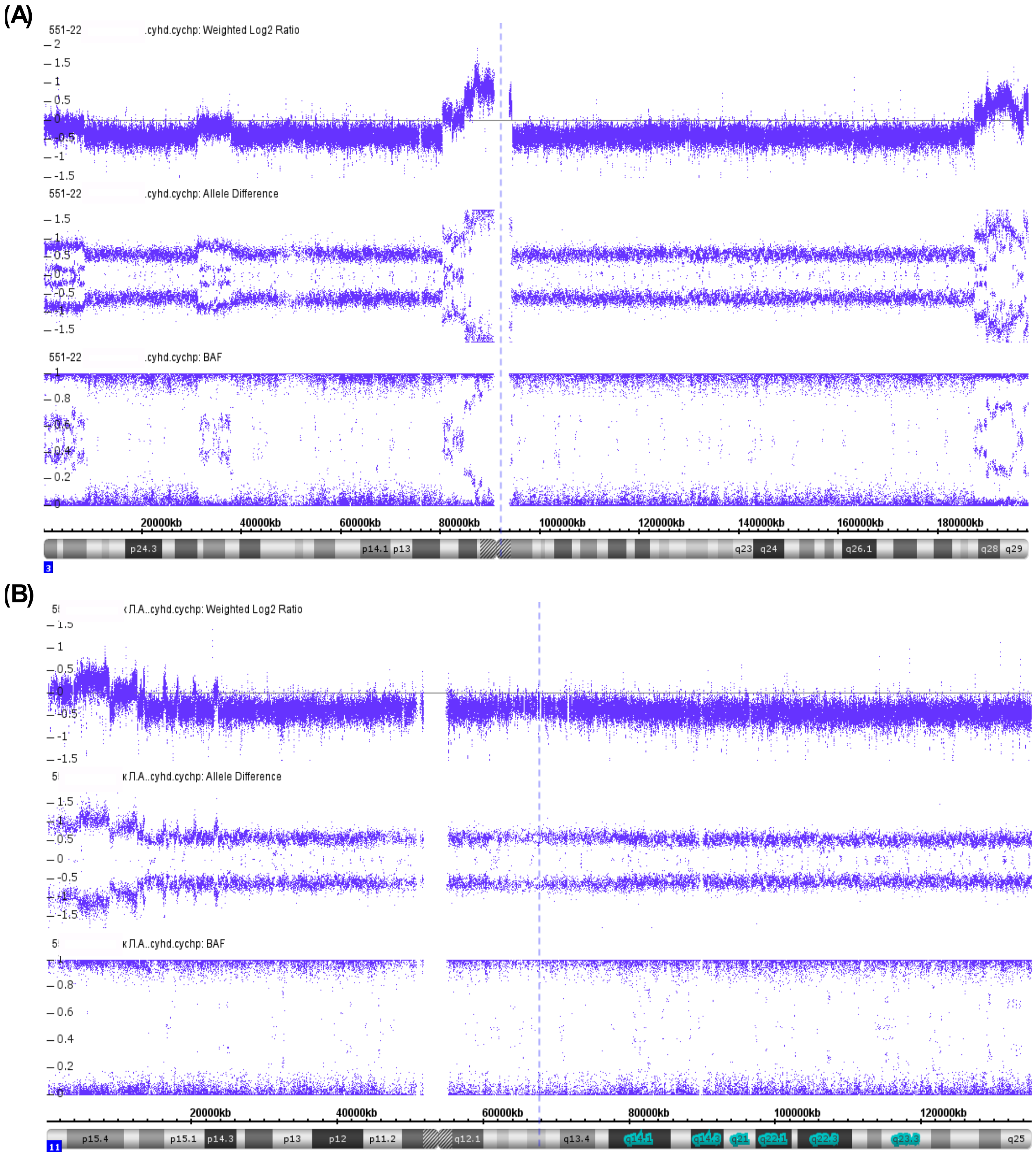

Supplementary figure 2. Representation of monosomy of chromosome 3 **(A)** and 11 **(B)**.

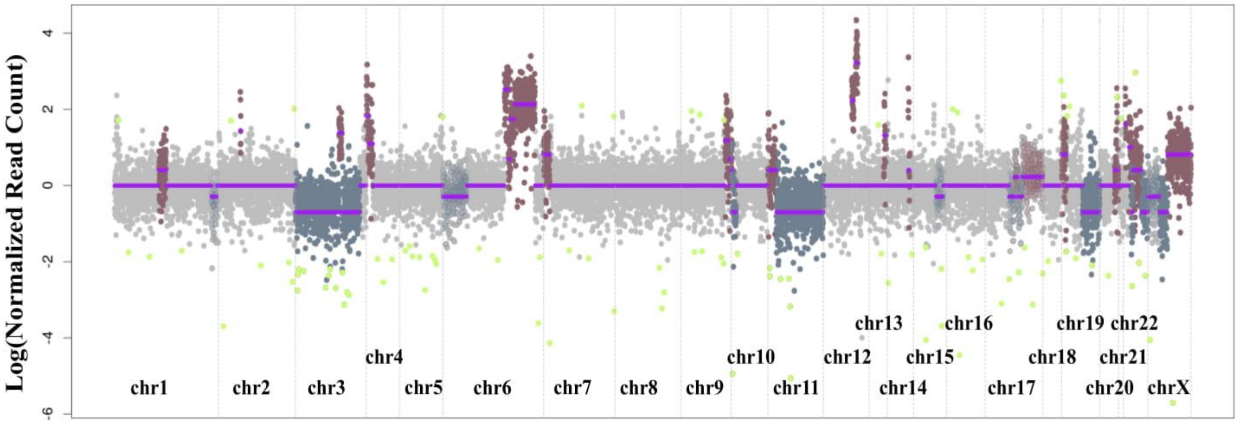

Supplementary Figure 3. Results of ONCOCNV analysis.

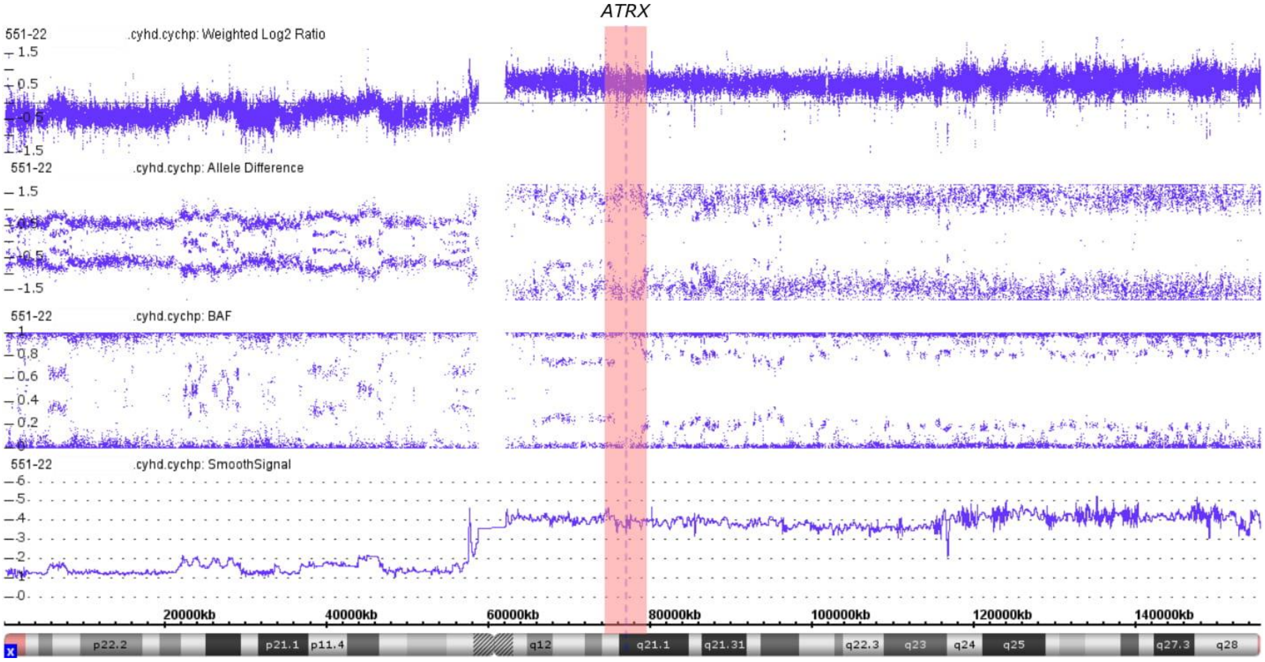

Supplementary Figure 4. Representation of chromosome X affected by loss in p arm and gain of q arm.

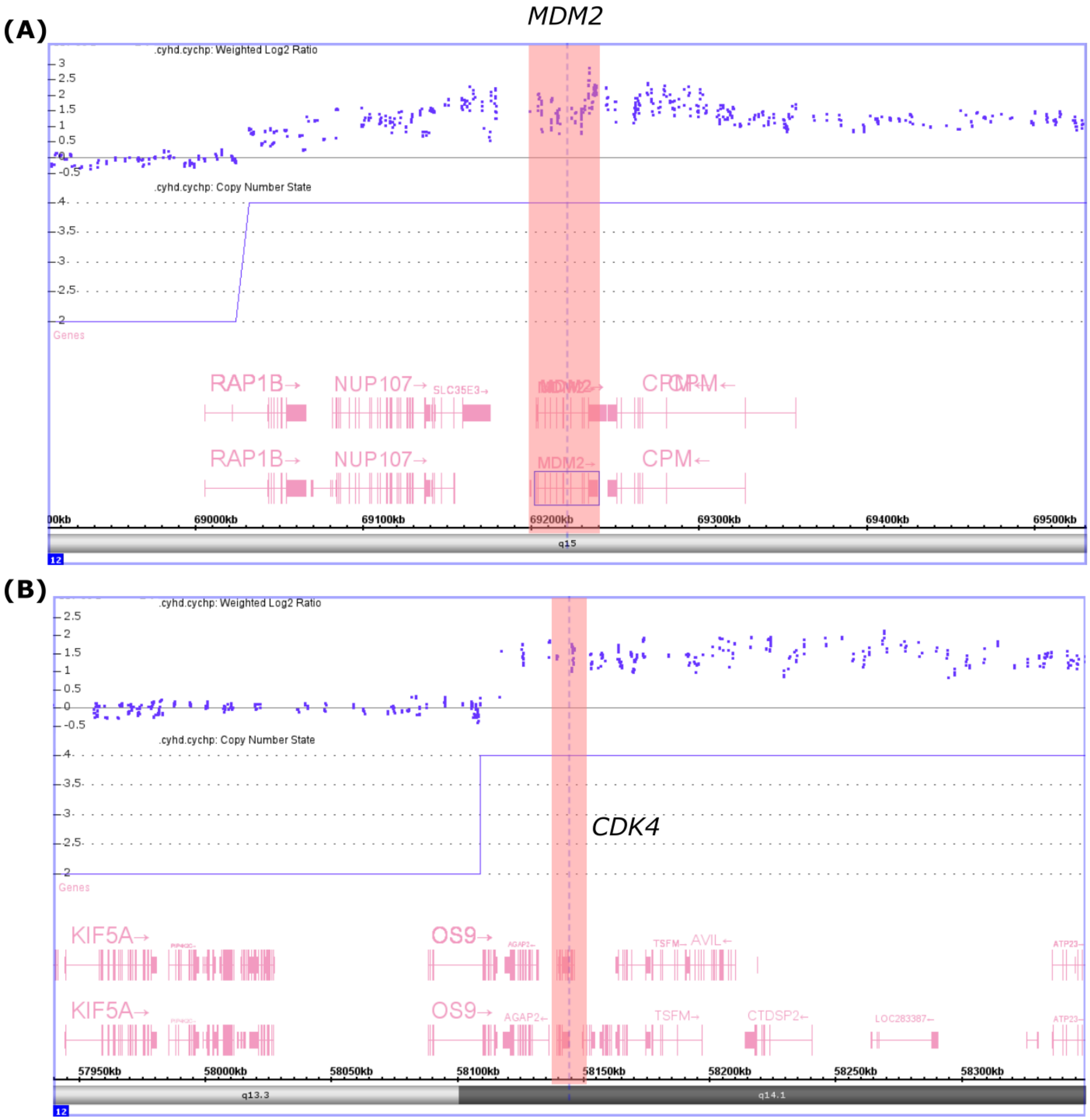

Supplementary figure 5. Representation of gains in regions 12q15 and 12q13.1-q14.1 that harbor MDM2 and CDK4 genes. **(A)** MDM2 gene with copy number state of four. **(B)** CDK4 gene with copy number state of four.

Supplementary table 1. Regions with homozygous losses.

Regions with homozygous losses		
Coordinates according to ISCN 2016 nomenclature	Length of loss, bp	Gene
arr[GRCh37]3p14.2(60286791_60291875)x0	5084	
arr[GRCh37]6q13(74590397_74601724)x0	11327	
arr[GRCh37]7q36.2(154393060_154402859)x0	9799	
arr[GRCh37]10p12.1(27625952_27688513)x0	62561	*PTCHD3* (NM_001034842)
arr[GRCh37]11q24.2(124078064_124080113)x0	2049	
arr[GRCh37]14q21.1(41615107_41657445)x0	42338	
arr[GRCh37]14q32.33(106890795_106918044)x0	27249	
arr[GRCh37]14q32.33(106530534_106667035)x0	136501	
arr[GRCh37]15q24.3(76891728_76895066)x0	3338	
arr[GRCh37]22q11.21(19568988_19573159)x0	4171	
arr[GRCh37]Xq23(115751422_115751430)x0	8	
arr[GRCh37]Xp22.33(1459624_1460944)x0	1320	*IL3RA* (NM_002183)
arr[GRCh37]Xp22.33(377893_377908)x0	15	
arr[GRCh37]Xp21.1(31860933_31860942)x0	9	
arr[GRCh37]Xp21.1(31889158_31889417)x0	259	
